# A rare cause of unilateral hypoglossal nerve palsy: case report of intraneural ganglion cyst of the hypoglossal nerve and review of the literature

**DOI:** 10.1080/23320885.2019.1599288

**Published:** 2019-04-15

**Authors:** Jeremie D. Oliver, Antonio J. Forte

**Affiliations:** a Mayo Clinic School of Medicine, Rochester, MN, USA;; b Division of Plastic and Reconstructive Surgery and Robert D. and Patricia E. Kern Center for the Science of Health Care Delivery, Mayo Clinic, Jacksonville, FL, USA

**Keywords:** Ganglion cyst, hypoglossal nerve, cranial nerve palsy

## Abstract

Benign lesions of the soft tissue arising in the periarticular space, such as a ganglion cyst, can cause compression of adjacent nerve fascicles passing in the nearby joint space. Intraneural ganglion cysts involving the cranial nerves are particularly rare, with only a few previous cases reported in the literature.

## Introduction

Ganglion cysts causing hypoglossal compression are rare. They are typically found in peripheral nerves near tendon sheaths, often near the carpal tunnel, or in the knee region near the fibular head [1–3]. Cranial nerves are rarely affected by intraneural ganglion cysts [3]. These cysts can present clinically by causing compression of the adjacent nerve fascicles, resulting in pain, paresthaesia, weakness, muscle denervation, and atrophy [1]. Significant clinical findings to be expected from an intraneural ganglion cyst of the hypoglossal nerve include unilateral tongue deviation and atrophy on the affected side, as well as potentially slurred speech or compression of nerves of the jugular foramen [4–7]. The present literature documents only four cases being reported [4–7]. We report an extremely rare case of a patient with a hypoglossal cystic lesion. The aim of this report is to present our surgical approach to treatment and to compare our findings with previous reported cases of unilateral hypoglossal nerve palsy, highlighting the importance of an intraneural (or extraneural) ganglion cyst in the differential diagnosis of such.

## Case report

**Figure 1.**
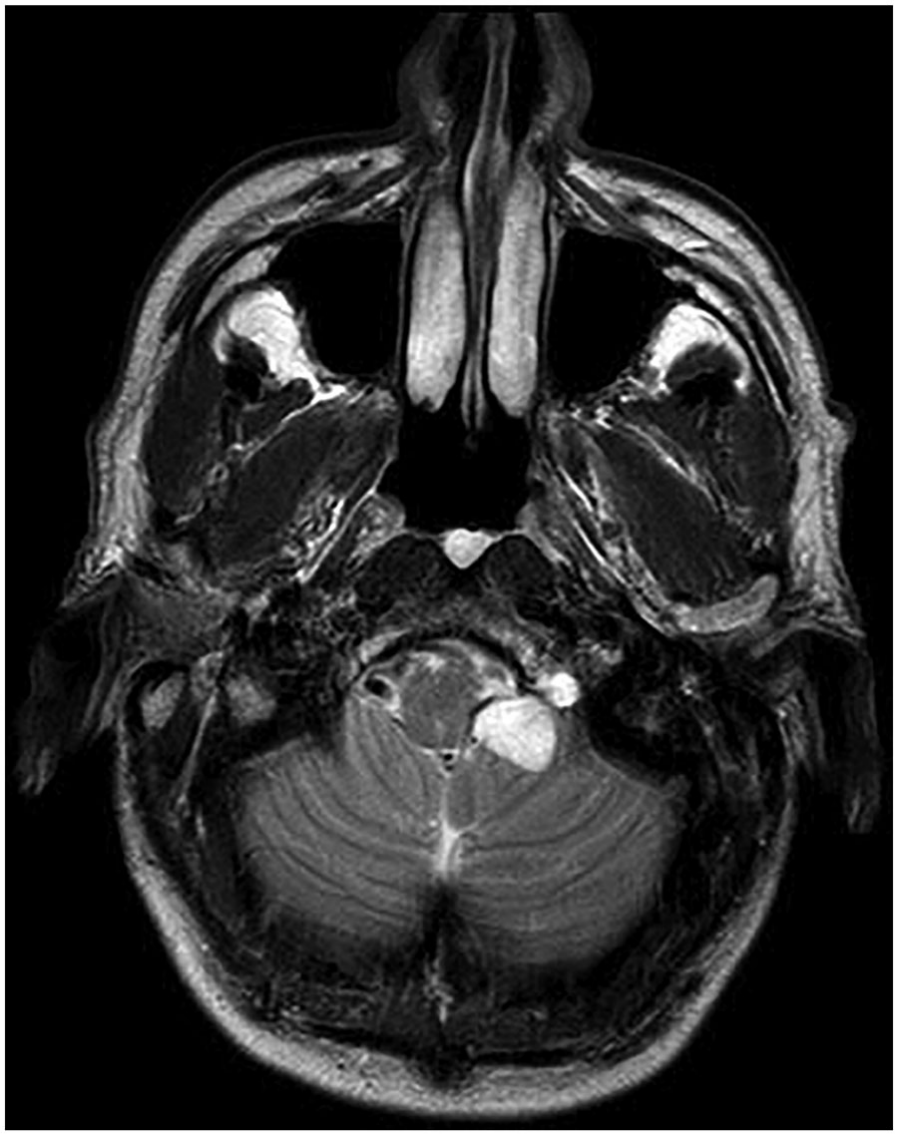
Axial T2 weighted magnetic resonance image (MRI) demonstrating lesion in the left hypoglossal foramen being T2 hyperintense with no gadolinium contrast enhancement.

**Figure 2.**
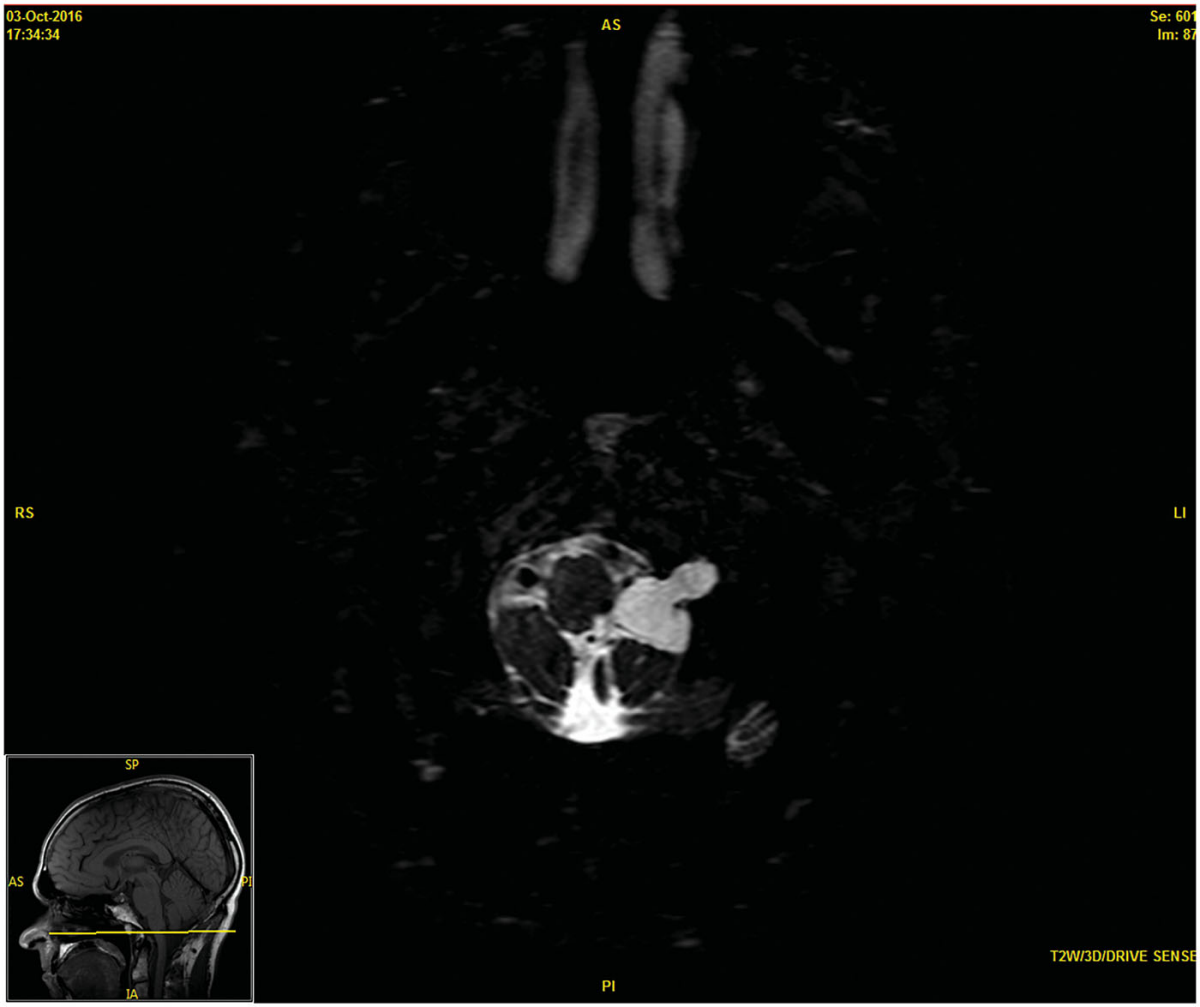
Follow-up T2 weighted 3D magnetic resonance image (MRI) 12 months after initial presentation demonstrating compression at the level of the brainstem.

A 56-year-old male presented to our department with a history of seven years of left tongue weakness and progressive atrophy. The patient noted, in the context of eating, that he seemed to be biting his tongue more on the left-hand side. Previous magnetic resonance imaging (MRI) studies performed at an outside institution revealed a cystic structure with T2 hyper intense signal centred on the hypoglossal nerve and hypoglossal canal. No intervention was performed at that time. Follow-up imaging several years later clearly demonstrated a progressively enlarging lesion in the left hypoglossal foramen being T2 hyperintense with no gadolinium contrast enhancement (Figure 1). Ultimately MRI studies one-year later showed substantial expansion of the lesion to approximately 2.5cm (Figure 2). Further CT imaging at that time demonstrated widening of bone in the region of the hypoglossal canal. Brainstem compression was notable, as well as hypoglossal and cerebellar tonsillar compression. At the time of meeting the patient, surgery was offered.

## Surgical approach

Surgical resection of the hypoglossal nerve lesion included a left sub-occipital craniotomy and C1 laminectomy to facilitate the supracondylar far lateral left-sided approach. The hypoglossal canal was not drilled out itself however the approach was used to best visualise the lesion. Intraoperative neural monitoring performed on CNX, XI, and XII on the left side, including somatosensory evoked potential (SSEP) monitoring, yielded no changes during resection.

A midline incision was made along the avascular plane down to the C1 and C2 region to expose the sub-occipital region. The sub-occipital region was eccentrically exposed to the left side. A sub-periosteal dissection was used over C1 to get out to the vertebral groove, and the skin and musculocutaneous flap were held with self-retaining retractors. A matchstick bit was used to drill troughs in C1 to facilitate its removal in one piece. A central sub-occipital craniotomy was further exposed (same configuration as Chiari decompression) and extended this over to left side. Drilling of the jugular tubercle was performed with a 3-diamond bit over the top of the supracondylar region in addition to drilled C1 flush to the pedicle. The dura was opened in a C-shaped fashion based on the left jugular tubercle to expose the tumour. The arachnoid space was subsequently opened separately, wherein was identified the cystic tumour of CNXII filling the foramen of Lushka. Initially investigation of the cyst revealed nerve rootlets over the back side along the vertebral artery. The vertebral artery was dissected away from the cyst as well as the spinal portion of the spinal accessory nerve. The mass itself was found to be mostly cystic, decompressing quite easily. Working around the margins of the extra-foraminal portion of the tumour, all nerve rootlets were dissected away, and ultimately removed the tumour completely. Intermittent stimulation (up to 3 mA) of the capsule was performed to ensure no functional nerve roots were being taken. Interestingly, the foramen of Lushka was expanded, and given the position, we were able to look directly out it. Mobilisation of the distal portion of the capsule allowed for complete removal out of the foramen without drilling it out. It is notable that approximately three nerve rootlets of the hypoglossal nerve were preserved in the inferior aspect of the canal. Continued aggressive removal of tumour was performed until there was no evidence of further tumour. The jugular foramen contents were intact without any evidence of disruption. The spinal portion of the spinal accessory nerve was intact.

**Figure 3.**
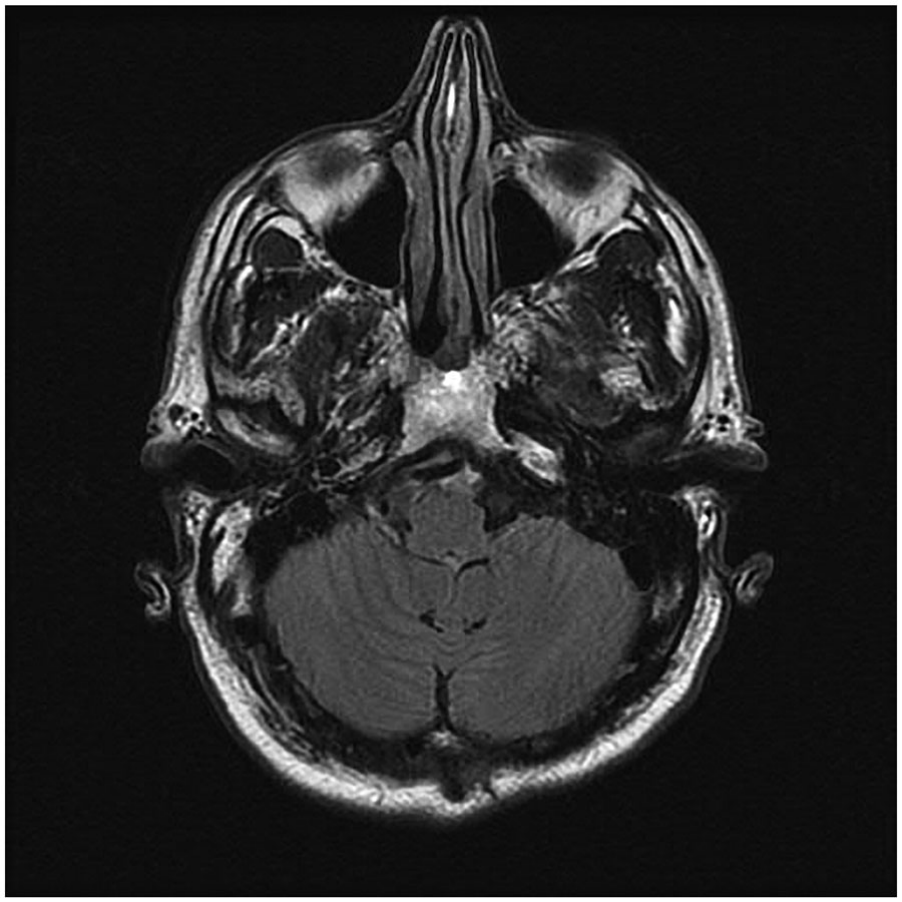
Post-operative (4 months) T2 flair axial magnetic resonance image (MRI) showing absence of lesion.

The intraoperative as well as the immediate postoperative course were uncomplicated. At the time of dismissal 2 days later he was ambulating independently and tolerating an oral diet. Final pathology returned a ganglion cyst of the hypoglossal nerve. Follow-up visit at 4-months postoperatively was completely benign, with revealed absence of ganglion cyst on MRI (Figure 3).

## Discussion

Unilateral hypoglossal nerve palsy has been reported in both benign lesions of the intra-articular facet joints disrupting nerve fascicles travelling through the joint space [8–10], as well as intraneural ganglion cystic lesions involving the nerve itself [4–7]. Detailed review of presenting signs and symptoms of both extraneural and intraneural ganglion cysts of the hypoglossal nerve reported to date demonstrated some unique aspects pertinent to each, respectively. Giordano et al reported a case of extraneural ganglion cyst, in which case the patient presented with a five-month history of headache and unilateral tongue weakness [10]. Similarly, Elhammady et al reported a case including a several month history of jaw, face, and neck pain on the side of the extraneural ganglion cyst [9]. The head and neck pain present in these two cases is unique to the extraneural cysts, as all five cases (including our case) of intraneural cysts did not present with pain in the head and neck region. Also unique to the extraneural cyst cases was weak pharyngeal reflex (reported in one case) and dysphagia (reported in all three cases). All reported intraneural ganglion cysts of the hypoglossal nerve have involved only the left hypoglossal nerve. The previous four cases reported have all presented with mild-to-moderate slurred speech (although ours did not present with any speech difficulty). All cases have demonstrated progressive hemiatrophy of the tongue upon presentation. Weighted imaging studies have demonstrated iso- or zero enhancement of the cyst in T1, and hyper-enhancement in T2 in all reported cases, both extraneural and intraneural. Morphologically, the cystic masses described in all previous accounts have been relatively homogeneous overall, presenting as gelatinous, “jelly-like”, easily-compressible, bluish masses upon resection. All cases of intraneural and extraneural ganglion cysts of the hypoglossal nerve have presented in adult patients, from age 51 to 70 years (see Table 1).

**Table 1.** Previously reported ganglion cysts involving the hypoglossal nerve.

	Intraneural					Extraneural		
Characteristic	Bilgin-Freiert et al. 2015 [4]	Gambhir et al. 2011 [6]	Nonaka et al. 2010 [5]	Baldauf et al. 2005 [7]	Present Case	Giordano et al. 2012 [10]	Elhammady et al. 2009 [9]	Mujic et al. 2003 [8]
Age (years)	Not reported	70	54	51	56	54	67	52
Sex	M	M	M	M	M	F	F	M
Laterality	Left	Left	Left	Left	Left	Left	Left	Left
Symptoms	Slurred speech	Progressive left hypoglossal nerve palsy, mildly slurred speech	Swallowing disturbance, hoarseness, & worsening shoulder weakness	6-mo hx of slurred speech	Palatal weakness, numbness	5-month history of headache and weakness of the tongue	3-mo hx of left jaw & facial pain, neck discomfort, difficulty w/ speech & swallowing	3-wk hx of dysarthria & difficulty swallowing
Signs	Left-sided tongue atrophy	Left-tongue haemiatrophy, tongue deviation (left) and moderate atrophy	Left tongue atrophy (7-yr hx)	Left tongue hemiatrophy	Left tongue atrophy (7-yr hx)	Hypoglossal nerve palsy and a weak pharyngeal reflex on the left.	Left tongue hemiatrophy & gait ataxia	Left tongue hemiatrophy
Imaging (MRI)	T1: no enhancement; T2: high	T1: iso, no enhancement; T2: high	T1: no enhancement; T2: high	T1: iso, no enhancement; T2: high (CT: hypoglossal canal enlargement)	T1: no enhancement; T2: high (CT: widening of bone in the region of the hypoglossal canal; brainstem, hypoglossal, and tonsillar compression)	T1: no enhancement; T2: high	T1: iso, no enhancement; T2: high	T1: no enhancement; T2: high
Surgical Approach	Transcondylar	Stereotactic suboccipital craniotomy	Transcondylar suboccipital	Far-lateral	Supra-condylar far-lateral	Lateral suboccipital	Juxtacondylar	Far-lateral
Intraoperative Findings	Gelatinous	Jelly-like, bluish-color	Gelatinous, multicystic	Gelatinous	Gelatinous, cystic (easily decompressed)	Gelatinous	Jellylike, amorphous mucoid	Clear, jellylike contents
Location	Intradural	Intradural	Intradural	Extra- & intradural (intraneural)	Intradural (filling the foramen of Lushka)	Intradural	Extradural	Extra- & intradural atlantooccipital joint
Final Pathology	Intraneural ganglion cyst of the hypoglossal nerve	Intraneural ganglion cyst of hypoglossal nerve	Similar to intraneural ganglion cyst (fragments of nerve w/ intervening strips of fibrous tissue; surrounding fibrous tissue & adherent nerve fibres wrapping around tumour)	Intraneural ganglion cyst (nerve fibres infiltrated & surrounded by myxoid connective tissue)	Intraneural ganglion cyst of the hypoglossal nerve, with projecting nerve rootlets encompassing the vertebral artery (dissected out)	Extraneural intradural bilobate ganglion cyst of the atlantooccipital joint compressing the hypoglossal nerve	1 yr: tongue paralysis persisted	Synovial cyst (synovial substance from the atlantooccipital joint infiltrated the nerve)

We present a case of intraneural involvement of a cystic lesion within the hypoglossal nerve, which constitutes only the fifth reported case to date. While these cases are often managed primarily by our neurosurgical colleagues, it is crucial for consulting plastic surgeons to have a thorough understanding of skull base pathology in order to effectively collaborate. We suspect the origin of the cyst to have been the occipital condylar joint. Given the novel intra-dural location of this patient’s lesion filling the foramen of Lushka, our approach involved a lateral sub-occipital exposure, combining a left sub-occipital craniotomy and C1 laminectomy to facilitate the supracondylar far lateral left-sided approach. This approach provided excellent exposure of the mass, thereby allowing complete tumour removal without risk of instability. Unique to this case, CT imaging demonstrated widening of the bone in the region of the hypoglossal canal, as well as brainstem, hypoglossal, and tonsillar compression from the cystic mass. Additionally, this was the only reported case to have presented with palatal weakness and numbness. From an operative standpoint, our patient’s intraneural ganglion cyst presented a unique challenge for removal, with projecting nerve rootlets encompassing the vertebral artery, which were dissected away. Peripheral nerve ganglion cysts demonstrate a variable recurrence rate, often thought to be due to incomplete removal of the lesion [1–3]. We feel that the aggressive microdissection performed in this case aided in the complete resection of the intraneural cyst without residual pathology seen at 4-month follow-up MRI. While complete resection of intraneural pathology can be obtained through meticulous dissection at the site of involvement, it should be noted (and discussed with the patient) that complete removal of intraneural ganglion cysts of the hypoglossal nerve does not restore motor function to the atrophic tongue on the affected side, as evidenced in this case and the others reported [4–6,9].
